# Theoretical background of the game design element “chatbot” in serious games for medical education

**DOI:** 10.1186/s41077-025-00341-7

**Published:** 2025-03-12

**Authors:** Alexandra Aster, Arietta Lotz, Tobias Raupach

**Affiliations:** Institute of Medical Education, University Hospital Bonn, Bonn, Germany

**Keywords:** Self-determination theory, Game design elements, Chatbot, Serious game, Medical education

## Abstract

**Background:**

The use of virtual patients enables learning medical history taking in a safe environment without endangering patients’ safety. The use of a chatbot embedded in serious games provides one way to interact with virtual patients. In this sense, the chatbot can be understood as a game design element, whose implementation should be theory driven and evidence based. Since not all game design elements are already connected to theories, this study aimed to evaluate whether the game design element chatbot addresses the need for autonomy rooted in the self-determination theory.

**Method:**

A cross-sectional study was conducted to compare two distinct chat systems integrated in serious games with one system being an open chatbot and the other system being a constrained chat system. Two randomized groups of medical students at a German medical school played one of two serious games each representing an emergency ward. The data collected included both objective data in terms of students’ question entries and subjective data on perceived autonomy.

**Results:**

Students using the open chatbot generally asked significantly more questions and diagnosed significantly more patient cases correctly compared to students using a constrained chat system. However, they also asked more questions not directly related to the specific patient case. Subjective autonomy did not significantly differ between both chat systems.

**Conclusion:**

The results suggest that an open chatbot encourages students’ free exploration. Increased exploration aligns with the need for autonomy, as students experience freedom of choice during the activity in terms of posing their own questions. Nevertheless, the students did not necessarily interpret the opportunity to explore freely as autonomy since their subjectively experienced autonomy did not differ between both systems.

**Supplementary Information:**

The online version contains supplementary material available at 10.1186/s41077-025-00341-7.

## Background

Serious games are increasingly used in medical education [1–3], but the optimal design of these games has not been completely established yet. In particular, the link between theoretical underpinnings and game design elements is not clear yet, although being fundamental [4]. This gap means that serious games may be less effective in achieving learning goals [4]. This study aimed to examine two different types of chatbots embedded in serious games, considered through the lens of self-determination theory, to determine the impact on learning behavior and students’ perceived autonomy.

### Training of history-taking skills in medical education

History taking contributes about 76% to the final medical diagnoses made by physicians [5]. In a study conducted with medical students, 43 out of 60 first-year medical students (71.7%) who diagnosed a simulated patient correctly made the correct diagnosis directly after taking the medical history [6]. Thus, it appears that teaching history taking to medical students is of particular importance. A systematic review revealed that there is a plethora of interventions used to teach history taking [7]. The types of intervention ranged from instructional approaches (i.e., focus scripts, video tape, or online courses) to more sophisticated approaches (i.e., small-group workshops with role-play, simulated patients, real patients, or virtual patients) [7]. While different interventions are applied for teaching history taking, simulated patients (SPs) are still used most frequently [7, 8]. SPs provide a risk-free learning environment for students to improve their communication skills [8]. Since training SPs requires resources and they are also a limited resource themselves [9], another efficient method of standardized training has emerged with virtual patients (VP) [10, 11]. VPs are a secure, reliable, and valid learning resource offering the opportunity of repeated exposure to the same potentially complex scenarios difficult to replicate in real life [11]. The authenticity of VPs depends on three aspects: the learner’s perception of the story surrounding the VP, the format, and the quality in which the VP is presented [12]. It is already shown that VPs can be developed emotionally responsive [13], which is relevant for training history taking. One way to access VPs during history taking is through chatbots [13, 14], which appear to be a learning resource largely equally effective as controls [14, 15]. A chatbot can be best defined as a computer program imitating human conversation when addressed through written or spoken language [16].

### Theoretical underpinnings of serious games as training environments

Serious games, defined as games whose primary aim is reaching a learning goal and not solely inducing fun or enjoyment [17], offer a learning environment in which VPs can be usefully integrated. An abundance of serious games already found entrance into health professions education and seem to be as effective as or more effective than not only control conditions such as traditional or digital learning formats but also other types of serious games or gamification [1]. Besides the effectiveness in terms of improved learning outcomes, serious games can also enhance the motivation and engagement of the players [2, 3] and should therefore be designed according to motivational theories. One such motivational theory frequently used in the design of serious games is the *self-determination theory* (SDT) [18]. According to the SDT, the three basic psychological needs for autonomy, competence, and relatedness have to be addressed to lead to intrinsic motivation [18]. The need for autonomy relates to the feeling of acting volitional according to one’s own will with perceived decision freedom enabling the choice between different kinds of action [19, 20]. The need for competence is addressed by the feeling of being capable to meet a goal based on the effective execution of one’s own behavior [20], while the need for relatedness is addressed by a sense of belonging to a reference group [19]. In serious games, these needs are addressed by inherent game design elements, which are essential for games to be characterized as such [21]. Existing literature already examined the importance for game design elements to be based on theoretical underpinnings [4]. Some game design elements were already linked with the addressed need, for instance points or badges refer to the need for competence, avatars or meaningful stories refer to the need for autonomy, and teammates refer to the need for relatedness [19]. However, a considerable number of game design elements have not yet been matched to the SDT.

### Chatbots as a game design element

In this sense, a chatbot embedded in a serious game can also be understood as a game design element with unclear theoretical background. It is long known that an autonomy fostering learning environment during medical education not only enhances students’ autonomous motivation but also positively influences their perseverance as well as their interaction with patients [22]. Previous research has shown that an addressed need for autonomy is aligned with greater experienced curiosity in terms of exploration [23]. It can be assumed that providing users with the opportunity to freely select or enter their queries may address their subjective feelings of autonomy, as reflected in exploratory behavior. Following this line of thought, the game design element “chatbot” might be assigned to the need for autonomy from the SDT, especially under the included aspect of decision freedom [19]. Since internal game analytics should be evaluated to not compromise the players’ flow during the game experience [24], it is reasonable to use the chatbot entries as an operationalization for assessing exploration and therefore autonomy. For the purpose of this study, a chatbot in which questions can be formulated freely via free entries is referred to as open. Vice versa, a chatbot in which questions can be selected from a set of predefined questions is referred to as constrained.

### Research aim

This research’s overarching aim was to assess whether the need for autonomy stemming from the SDT can be linked to the serious games’ game design element “chatbot” and whether this association depends on the type of chatbot used. Therefore, two serious games presenting different history-taking systems with different degrees of freedom were compared. The need for autonomy was operationalized through medical students’ free exploration during history taking. It is assumed that an open chatbot that mimics a real-world situation by requiring self-formulated questions addresses students’ autonomy due to offering a free environment with the opportunity of decision freedom expressed through free exploration.

- H1: Students ask significantly more questions in an open chatbot compared to a constrained chat system.
- H2: Students ask significantly more irrelevant questions in an open chatbot compared to a constrained chat system.
- H3: Students report significantly more subjective feelings of autonomy in an open chatbot compared to a constrained chat system.

## Methods

The local Institutional Review Board at Göttingen Medical School approved this study in winter term 2023/2024 (application number: 8/9/23). All participants gave written informed consent beforehand.

### Study procedure

The study was conducted in a mandatory module for fourth-year undergraduate medical students covering the areas cardiology and pneumology at Göttingen medical school in winter term 2023/2024. All students attending the module were invited to voluntarily participate in the study, but participation was not mandatory. The module comprised four sessions each lasting 90 minutes. However, only the data collected during the first session were relevant for this study, as it was the first time students interacted with the serious games, ensuring that the data were not biased by familiarity with the game. Students were randomly assigned to one of two study groups. One group engaged in on-site gameplay of the serious game EMERGE [25], representing the constrained chat system, while the other group simultaneously played the serious game DIVINA [26] online, representing the open chatbot. Both serious games provided the students with the diseases ST-segment elevation myocardial infarction (STEMI), non-ST-segment elevation myocardial infarction (NSTEMI), musculoskeletal chest pain, and hypertensive crisis, while DIVINA additionally provided the disease congestive heart failure. At the end of the first session, students were invited to participate in an evaluation.

### Serious game environments

Both serious games represent emergency departments with similar procedures within the games, although differing in their visual design as well as in their game structure. In both games, players take a patients’ medical history, order investigations, initiate treatments, and finally discharge the patient. For the present study, the focus is only on the manner how the medical history taking takes place. In the serious game EMERGE, players use the constrained chat system by choosing from a long menu of 70 predefined questions. Precisely, students enter specific letters or words included in their sought question to which the long menu proposes suitable questions including the entered letters or words. Please refer to Middeke, Anders [25] for further information on the design of EMERGE. Contrary to EMERGE, the serious game DIVINA does not provide predefined questions for medical history taking, but students have to phrase questions themselves in an open chatbot. The chatbot refers to a script-based system and provides answers based on a system that draws on information about the specific virtual patient and their symptoms. Please refer to Aster, Hütt [26] for further information on the design of DIVINA. In both serious games, students are not limited in the number of questions for taking a sufficient medical history.

### Data collection and preparation

#### History data

All qualitative history data gathered in both games were quantified first. To do so, a checklist was developed in collaboration between a physician specialized in the field of cardiology and a psychologist. The physician contributed medical expertise and ensured content accuracy, while the psychologist focused on assessing psychometric properties of the checklist. These two authors used the checklist to independently and blindly score all history-taking data for both serious games. For the sake of uniformity, the same checklist was used for all diseases. The data were quantified in the way that all questions were scored irrespectively of the received answer, and each question was rated once regardless of reformulations. More precisely, it was irrelevant whether students received a sufficient and satisfactory answer; the questions were evaluated independently of the received answers. Depending on the medical relevance, questions were scored with 1 or 2 points. Overall, a total of 49 points could be achieved. The checklist oriented towards the SAMPLER/OPQRST scheme [27] and contained the following areas: “basic patient-related data”, “current reason for consultation”, “specific somatic anamnesis” (subdivided in “current complaints and development” and “focused pain anamnesis”), “general somatic anamnesis” (containing “past medical history”, “vegetative anamnesis”, “risk factors”, in particular “cardiovascular risk factors”), as well as “family and social anamnesis”, and “orienting psychiatric anamnesis”. The complete checklist can be found in Supplementary 1.

#### Questionnaires

The evaluation consisted of the subscale for “perceived choice” from the *Intrinsic Motivation Inventory* (IMI) [28] and the *General Self-Efficacy Short Scale* (German: Allgemeine Selbstwirksamkeit Kurzskala, ASKU) [29]. Both questionnaires are reliable and validated measuring instruments [28, 29]. The IMI was chosen to measure the intrinsic motivation of students, while the ASKU was chosen to measure self-efficacy, which can be considered as related to the SDT. According to Bandura [30], self-efficacy implies that a person has the belief to successfully master a situation by performing the necessary behavior. Moreover, self-efficacy has already been found to be an underlying construct for gamification [31].

### Data analysis

#### History data

The history data were analyzed according to the following procedure: In a first step, the interrater reliability for both authors was assessed. All analyses were conducted utilizing a mean rating score derived from the assessments provided by both reviewers for each serious game, hereinafter referred to as “history score”. Since normal distribution of the data was not given, nonparametric statistical methods or methods that are not affected by violation of this assumption were chosen. For each statistical procedure, the corresponding effect size was conducted and reported.

Prior to hypothesis 1, descriptive statistics about the serious games were evaluated, and a chi-squared test was conducted to assess differences in the number of correctly diagnosed cases between the serious games.

For hypothesis 1, which states that students asked significantly more history questions in an open chatbot (i.e., DIVINA) than in a constrained chat system (i.e., EMERGE), a Mann–Whitney *U*-test comparing the absolute number of questions between the two groups was conducted.

A hierarchical sequence of steps was followed to evaluate hypothesis 2, which states that students significantly asked more irrelevant questions in an open chatbot compared to a constrained chat system. Firstly, a Mann–Whitney *U*-test for comparing the achieved history scores between the two serious games was performed. Following this, a regression analysis to examine the relation between the number of questions asked and the achieved history scores for each serious game was performed. The irrelevance was defined as a ratio of the achieved history score and the number of questions asked for each chat protocol separately. A ratio < 1 represents that more questions were asked than points were achieved implying the presence of more irrelevant questions. Vice versa, a ratio > 1 implies less irrelevant questions since a higher history score was achieved with less questions asked, in a sense that the history score exceeds the number of questions. For examining the hypothesis, a final Mann–Whitney *U*-test comparing the ratios between the two groups was conducted.

#### Questionnaires

The questionnaire data were analyzed for answering hypothesis 3 stating that students reported significantly higher subjective autonomy feelings after playing DIVINA compared to EMERGE. Both questionnaires were analyzed according to its guidelines before a mean value comparison was conducted. Since these data were not normally distributed, Mann–Whitney *U*-tests were carried out.

## Results

### History data

*N* = 154 fourth-year medical students consented to have their data entries in the serious games analyzed. Since all data were recorded anonymously, no further statements and conclusions about the population could be made except them being fourth-year students at a German medical school. Interrater reliability was computed for both serious games using the intraclass correlation coefficient (ICC), resulting in an ICC of 0.890 for DIVINA and an ICC of 0.939 for EMERGE. According to Cicchetti [32], both coefficients can be interpreted as very good agreements.

Only chat protocols containing at least one question were deemed valid. This led to 249 valid chat protocols stemming from DIVINA (4 of 254 initial chat protocols had to be excluded) and 456 valid chat protocols stemming from EMERGE (62 of 518 initial chat protocols had to be excluded). Students correctly diagnosed 162 patient cases (65%) in DIVINA and 236 patient cases (52%) in EMERGE (*χ*^2^ (1) = 13.025, *p* < 001). Generally, the number of questions asked per chat protocol in DIVINA ranged from 3 to 57 (*Mdn* = 13) and in EMERGE from 1 to 40 (*Mdn* = 9). Students asked significantly more questions in DIVINA than in EMERGE, *U* = 37,980.000, *p* < 0.001, *r* = 0.27, although with a weak effect size [33].

**Fig. 1:**
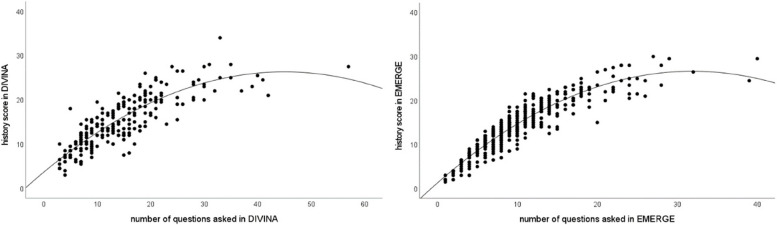
Scatterplot of the polynomial regression for both serious games

For evaluating whether students asked a higher number of irrelevant questions in an open chatbot, several analyses were conducted. In a first step, it was found that the achieved history scores did not differ significantly between DIVINA (*Mdn* = 14.5) and EMERGE (*Mdn* = 14), *U* = 51,766.00, *p* = 0.053. In a next step, it was examined whether the number of questions asked was related to the achieved history score. A polynomial regression was conducted for each serious game, since the assumption of linearity required for performing a linear regression was not met. The models were significant for both serious games, DIVINA (*F*(2248) = 307.44, *p* < 0.001) and EMERGE (*F*(2455) = 1508.84, *p* < 0.001). All specific parameters for both serious games can be found in Table 1. The scatterplot of the polynomial regressions for the relation between the number of questions asked and the achieved history score can be found in Fig. 1.

**Table 1:** Overview of the parameters of the polynomial regression

		*R*^*2*^	Adjusted *R*^2^	*β*	*SE*	*t*	*p*
DIVINA		0.714	0.712				
	Linear term			1.008	0.068	14.72	< .001
	Squared linear term			− 0.011	0.002	− 7.07	< .001
EMERGE		0.869	0.869				
	Linear term			1.593	0.048	32.91	< .001
	Squared linear term			− 0.025	0.002	15.63	< .001

**Fig. 2:**
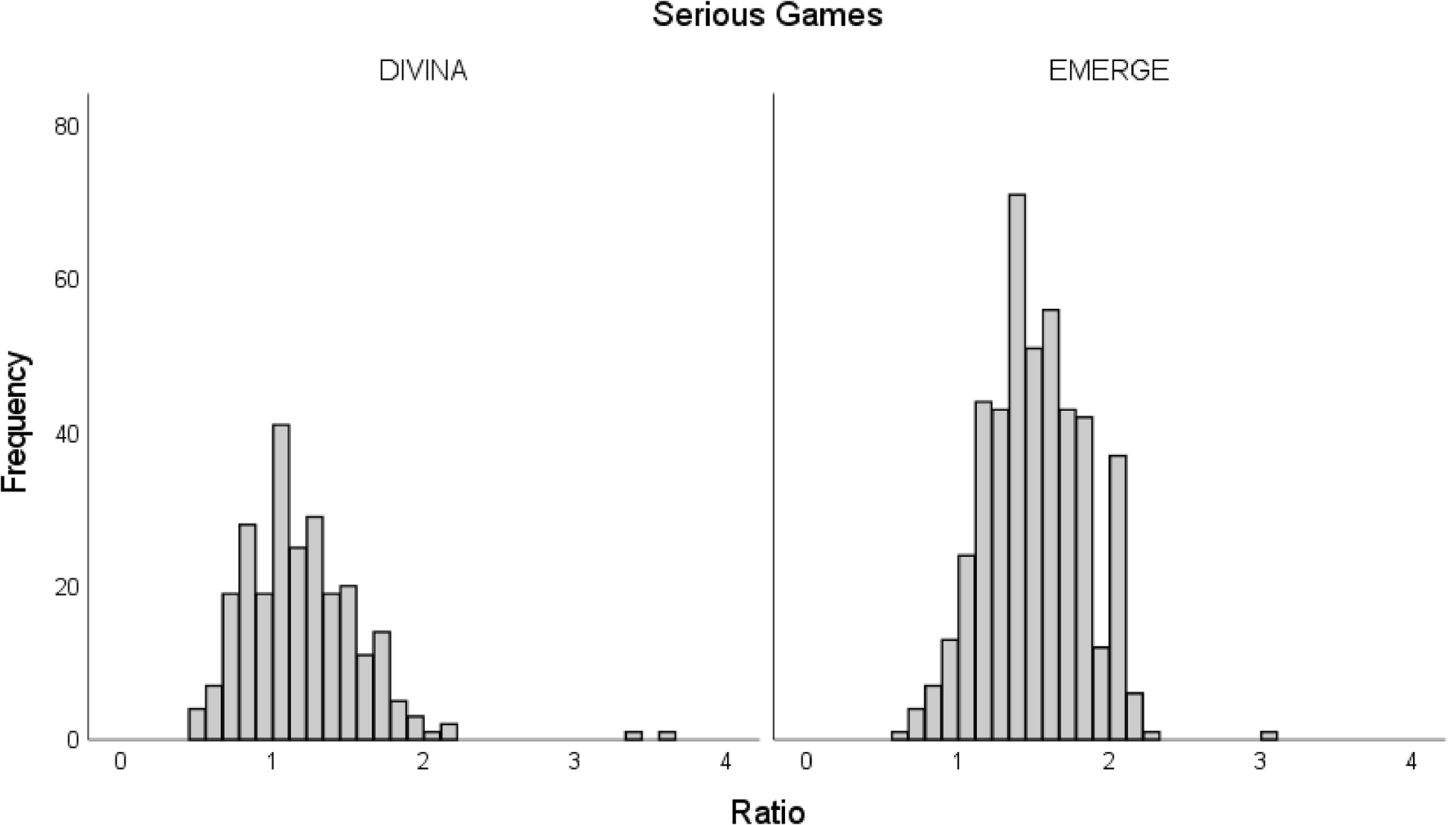
Distribution of the frequencies of the ratios

The subsequent Mann–Whitney *U*-test using the ratio showed that significantly more points were achieved by asking less questions in EMERGE (*Mdn* = 1.5) than in DIVINA (*Mdn* = 1.13), *U* = 28,367.000, *p* < 0.001, *r* = 0.41 with a moderate effect size [33]. The indicated medians refer to the abovementioned ratio of which the distribution of frequencies can be found in Fig. 2. Supporting the hypothesis, the lower ratio indicates a tendency to ask more irrelevant or not expedient questions in DIVINA.

### Subjective autonomy measures

Overall, *N* = 81 (*n* = 44 DIVINA, *n* = 37 EMERGE) students participated in the questionnaire of which *n* = 41 data sets could be analyzed for DIVINA and *n* = 35 for EMERGE. Considering the subjectively experienced autonomy during the play of both games, the autonomy scale of the IMI showed no significant differences between the experienced autonomy in DIVINA (*Mdn* = 4.29) and EMERGE (*Mdn* = 4.43), *U* = 654.00, *p* = 0.507. An explorative analysis addressed the relationships between the autonomy scale and the ASKU, since the SDT and self-efficacy are already jointly used constructs in the design of serious games [34]. Across the two serious games as well as broken down for each serious game, no significant correlation was found. Moreover, no significant group difference between EMERGE and DIVINA was found for the ASKU*, U* = 627.00,* p* = 0.266.

## Discussion

### General discussion

This study aimed to determine whether the need for autonomy stemming from the self-determination theory can be understood as a theoretical basis for the game design element “chatbot”. For the purpose of this study, autonomy was operationalized by students’ free exploration during history taking as well as by subjective autonomy ratings.

Overall, students showed better objective results (i.e., correctly diagnosed patient cases) in DIVINA (open chatbot) than in EMERGE (constrained chat system) and gained a slightly higher history score without there being a significant difference. The serious games were similar in content and action possibilities; however, they differed in their specific appearance. Since our analyses focused solely on interactions within the chatbot rather than the entire serious game, we assume that these differences in appearance did not affect the results. The first hypothesis that more history-taking questions were asked in an open chatbot compared to a constrained chat system showed a significant difference albeit with a weak effect size. Thus, it can be assumed that students explored more during history taking when provided with the opportunity to ask self-developed questions. The results support the assumption that the opportunity to formulate questions on their own fosters students’ exploration as operationalized by the amount of questions. Nevertheless, the amount of questions asked was relatively small for both serious games. One possible explanation could be the internal setting of the serious games (i.e., an emergency department), which may prompted students to prioritize further investigations over asking additional history taking questions.

Based on the idea of hypothesis 1, the next logical step was to examine whether students did not only ask more questions but also asked more irrelevant questions in an open chatbot compared to a constrained chat system. Therefore, hypothesis 2 was driven by the assumption that more irrelevant questions were asked as a result of increased exploration. In this context, irrelevant questions are queries, statements, or nonconstructive inputs that are not directly focusing on the central aspects of the patient case in terms of furthering the medical treatment. Nevertheless, the questions do not necessarily have to be irrelevant in the medical sense. In line with the hypothesis, the analysis revealed a significant difference with a moderate effect, indicating that students tend to ask more irrelevant questions in an open chatbot. Since the open chatbot was sometimes unable to usefully reply to the initial question, students tried to handle it by reformulating their entry. This increased the number of questions but did not affect the score, as these questions were only scored in their initial version. It is already known that script- or rule-based chatbots show difficulties in understanding the input, demonstrated by a virtual patient mismatching approximately 40% of students’ entries with the appropriate response [11]. An upcoming area of interest is the attempt to use large language models (LLMs) for the simulation of virtual patients [35, 36]. Further studies could consider using LLMs and thereby assessing students’ perceived autonomy using sound research designs.

An explanation for the difference in the amount of irrelevant questions derives from the manner of how questions were asked. While students needed to formulate their own questions in accordance with the rule-based open chatbot, the constrained chat system presented all possible requested questions, from which students only needed to select. Moreover, the generally limited number of available questions could have led to less irrelevant questions in the constrained chat system. At the same time, in this scenario, opportunities for students to pursue their own line of inquiry were very limited. The moderate effect size suggests that students nevertheless did not chose the perfect amount of questions in the constrained chat system, although the long menu format already disclosed potential questions. Although the use of in-game analytics is a recommended approach in serious game research [24], it is worth noting that students’ actions are difficult to interpret without considering their intent. Future research should aim to capture students’ intent and merge these insights. By means of the ratio, results showed that neither in the open chatbot nor in the constrained chat system one question led to one point, which may have been also caused by the amount of irrelevant questions or entries. Generally, an explanation for the relatively low history scores might be that students are possibly not familiar enough with history taking. Further studies should address this idea by adding an intervention to the study design. Furthermore, it would be intriguing to calculate the number of questions required to reach a diagnosis and examine its accuracy. Doing so, it could be tested whether the statement that up to three-quarters of the diagnoses are already correct after taking a history also applies for history taking with VPs [5].

Besides the objective data, the subjective data gave important insights on the experienced autonomy during each history taking. The subjectively experienced autonomy did not significantly differ between both serious games. Together with the results of hypothesis 2, it can be concluded that although students did not subjectively feel more autonomous in an open chatbot than in a constrained chat system, they still asked more questions and subsequently got more diagnoses correctly in the open chatbot. It is assumable that the discussed limitations associated with an open albeit script-based chatbot may have negatively influenced students’ feelings of autonomy. Consequently, students felt rather forced than autonomous during the interaction with the script-based chatbot given the necessary reformulation of their questions. These assumptions are based on questionnaire data, and although questionnaires are a frequently used measuring instrument for assessing autonomy, this particular questionnaire might not have been a sufficient instrument for the present study. Future research should consider alternative approaches, such as focus groups, which may yield different insights. However, a meta-analysis on gamification found that in most of the included studies, taking part in gamified classes enhanced students’ perception of autonomy [37]. Nonetheless, in line with our results, the authors found studies where gamification did not lead to enhanced perceptions of autonomy [37].

### Limitations

The limitations primarily concern the generalizability of the results due to the used game environments as well as the used data analysis instrument. Both serious games simulate emergency departments, raising the question whether this setting with its time pressure is adequate for studying students’ history taking. Moreover, some among the studied diseases might have required more, and some perhaps needed less history taking due to the risk of serious deterioration or even life-threatening complications. It has to be considered whether other settings, such as a general practitioner’s practice, an outpatient clinic, or a normal ward, are more suitable for examining students’ history taking. Future studies could possibly examine these different settings and contextualize the medical history within the framework of other conducted investigations to clarify the role of history taking in order to provide better generalizability.

The predefined checklist used to rate the history data constitutes another limitation. The checklist was oriented towards the SAMPLER/OPQRST scheme [27] that is commonly used in emergency management and includes a section specifically related to pain. Not all included diseases manifested with pain; however, due to the structure of the serious games’ outputs, it was not possible to control for whether the virtual patient presented with pain. As a result, the entire checklist was applied across all patient cases to provide comparability and to do justice to students specifically asking for pain. Second, the checklist was used for all diseases without being specialized for some diseases. While this procedure enhanced the simplicity of the data preparation, it may have also led to biased history scores. Future research should use disease-specific checklists tailored to the presented symptoms and count redundant questions.

Due to the different amount of subjective and objective data, drawing any conclusions on possible correlations between them was not possible. Moreover, due to the lack of identifying data, it was not possible to match questionnaire answers with the respective objective data.

## Conclusion

Our research focused on the theoretical underpinning of the game design element “chatbot”. Two chatbot systems were compared to determine whether the need for autonomy stemming from the self-determination theory is addressed when using a chatbot. We observed more exploratory behavior favoring autonomy in student history taking with an open chatbot, but our measures of subjective student experience did not reflect that. Even though measuring instruments require reconsideration to confirm this assumption, our study yields initial proof that an open chatbot may address the need for autonomy as operationalized by students’ exploration behavior. In conclusion, open chatbots can be considered valuable tools for medical students to practice history taking. However, further research is needed to identify the specific characteristics of chatbots that contribute to fostering autonomy during their use.

## Supplementary Information

Supplementary Material 1. Checklist.

## Data Availability

The datasets used and/or analyzed during the current study are available from the corresponding author on reasonable request.
