# Supplementary material for: Theoretical background of the game design element “chatbot” in serious games for medical education
Source: Adv Simul (Lond). 2025 Mar 12;10:10. doi: 10.1186/s41077-025-00341-7 (PMC11905647; doi:10.1186/s41077-025-00341-7)
Supplement: Supplementary file 1 — Supplementary Material 1. Checklist. [file 41077_2025_341_MOESM1_ESM.pdf]

| Points | Questions |
|---|---|
| **Basic patient-related data** | |
| 1 | Family status, profession |
| 2 | Height & weight (risk factor for diabetes and hypertension; weight fluctuations) |
| **Current reason for consultation** | |
| 2 | What exactly brings you in today? / What can we do for you? |
| **Specific somatic anamnesis** | |
| | **Current complaints and development** |
| 2 | Temporal occurrence, rhythm, duration |
| 2 | Name and localization of the complaints |
| 2 | Quality, degree of severity, and course of complaints |
| 2 | Conducted actions and subjective theory of disease |
| | **Focused pain anamnesis** |
| 1 | "Are you experiencing any pain?" |
| 2 | Localization of pain (located / radiating) |
| 2 | Temporal occurrence and duration of pain (beginning, wavelike / pulsating) |
| 2 | Quality of pain (burning / dull) |
| 2 | Pain intensity (strength / extent) |
| 2 | Attendant symptoms (edemas, vertigo, nausea, syncope) |
| 2 | Pain provoking or analgesic circumstances |
| 2 | Influenceability of the pain |
| **General somatic anamnesis** | |
| | **Previous illnesses** |
| 2 | Cardiovascular, metabolic, pulmonary / bronchial, digestive and neurological diseases |
| 1 | Infectious diseases and mental / psychiatric conditions |
| | **Past medical history** |
| 1 | Hospitalization, operations, accidents |
| 2 | Allergies, drug intolerances |
| 2 | Current / past medication intake and nutritional supplements |
| 1 | Travel history / stays abroad |
| **Vegetative anamnesis** | |
| 1 | Appetite, thirst, weight changes, fever, night sweat, nausea |
| 1 | Cough, sputum, digestion, urination, sleep |
| **Risk factors** | |
| 1 | Alcohol and drug consumption |
| 1 | Dietary habits, exercise, visits to the doctor, (preventive) medical check-ups |
| **Cardiovascular risk factors** | |
| 2 | High blood pressure, diabetes, hypercholesterolemia, heart diseases, stroke |
| 2 | Pre-existing conditions in the family |
| 2 | Smoking habits, lack of exercise, overweight |
| **Family and social anamnesis** | |
| 1 | Family status, psychosocial stressors, medical power of attorney, living will, patient-related release from the duty of confidentiality |
| **Orienting psychiatric anamnesis** | |
| 1 | Current mental condition |
